# *Lmod2piggyBac* mutant mice exhibit dilated cardiomyopathy

**DOI:** 10.1186/s13578-016-0101-y

**Published:** 2016-06-04

**Authors:** Shuang Li, Kaiqi Mo, Hong Tian, Chen Chu, Shuna Sun, Lei Tian, Sheng Ding, Tong-ruei Li, Xiaohui Wu, Fang Liu, Zhen Zhang, Tian Xu, Ling V. Sun

**Affiliations:** State Key Laboratory of Genetic Engineering and National Center for International Research of Development and Disease, Fudan-Yale Center for Biomedical Research, Innovation Center for International Cooperation of Genetics and Development, Institute of Developmental Biology and Molecular Medicine, School of Life Sciences, Children’s Hospital of Fudan University, Fudan University, Shanghai, China; Cardiac Center, Children’s Hospital of Fudan University, Shanghai, China; Howard Hughes Medical Institute, Department of Genetics, Yale University School of Medicine, New Haven, CT USA; Shanghai Pediatric Congenital Heart Institute, Institute of Pediatric Translational Medicine, Shanghai Children’s Medical Center, Shanghai Jiaotong University, School of Medicine, Shanghai, China

**Keywords:** Dilated cardiomyopathy, Leiomodin2 mutant, Sarcomere, Intercalated discs

## Abstract

**Background:**

Leiomodin proteins, Lmod1, Lmod2 and Lmod3, are key regulators of the thin filament length in muscles. While *Lmod1* is specifically expressed in smooth muscles, both *Lmod2* and *Lmod3* are expressed in striated muscles including both cardiac and skeletal muscles. We and others have previously shown that *Lmod3* mainly function in skeletal muscles and the mutant mice display disorganized sarcomere. Lmod2 protein has been found to act as an actin filament nucleator in both cell-free assays and in cultured rat and chicken cardiomyocytes.

**Results:**

To better understand the function of *Lmod2* in vivo, we have identified and characterized a *piggyBac* (*PB*) insertional mouse mutant. Our analysis revealed that the *PB* transposon inserts in the first exon of the *Lmod2* gene and severely disrupts its expression. We found that *Lmod2*^*PB/PB*^ mice exhibit typical dilated cardiomyopathy (DCM) with ventricular arrhythmias and postnatal lethality. Electron microscope reveals that the *Lmod2*^*PB/PB*^ hearts carry disordered sarcomere, disarrayed thin filaments, and distorted intercalated discs (ICDs). Those ICDs display not only decreased convolutions, but also reduced electron-dense staining, indicating less ICDs component proteins in *Lmod2*^*PB/PB*^ hearts. Consistent with the phenotype, the expression of the ICD component genes, β-catenin and Connexin43, are down-regulated.

**Conclusions:**

Taken together, our data reveal that *Lmod2* is required in heart thin filaments for integrity of sarcomere and ICD and deficient mice exhibit DCM with ventricular arrhythmias and postnatal lethality. The *Lmod2*^*PB/PB*^ mutant offers a valuable resource for interrogation of pathogenesis and development of therapeutics for DCM.

## Background

The Leiomodin proteins, Lmod1, Lmod2 and Lmod3, are a subgroup of the Tropomodulin (Tmod) protein family and are regulators of the thin filament length in muscles [1–3]. While *Lmod1* is specifically expressed in smooth muscles, both *Lmod2* and *Lmod3* are expressed in striated muscles including both cardiac and skeletal muscles [4–6]. We and others have previously shown that *Lmod3* mainly functions in skeletal muscles [6–8] and *Lmod3* mutants exhibit muscle atrophy in fast fibers [6]. The mutant mice display disorganized sarcomere and the presence of nemaline bodies in skeletal muscles, a hallmark of the disease nemaline myopathy (NM), consistent with the finding that *LMOD3* is mutated in the NM patients [7].

Lmod2 protein has been found to act as an actin filament nucleator in both cell-free assays and in cultured rat and chicken cardiomyocytes [2, 5]. Overexpression of *Lmod2* results in the elongated thin filaments and knockdown exhibited disrupted sarcomere assembly in cultured cardiomyocytes [2, 5]. Furthermore, it has been shown that Lmod2 is an antagonist of Tmod1 in cardiomyocytes [2, 5]. Knockout mice of Tmod1 are embryonic lethal due to cardiac defects, and overexpression of *Tmod1* in the heart causes myofibril disorganization and dilated cardiomyopathy (DCM) [9–13]. However, the physiological function of *Lmod2* remains unknown. We hypothesized that the phenotype of loss of Lmod2 in mice might mimic that of the overexpression of its antagonist *Tmod1*, and the mutant mice are high likely to carry DCM.

Dilated cardiomyopathy is a common form of cardiomyopathies and the third most common inheritable heart disease [14]. DCM is diagnosed as dilated left ventricular accompanied by systolic dysfunction, less than 50 % ejection fraction (EF) [15]. These symptoms may be accompanied by other complications, including arrhythmias, coagulation, and congestive heart failure (CHF), which often lead to lethality [16, 17]. It has been estimated that DCM causes at least half of heart failures and less than 50 % of the DCM patients survive beyond year 5 after the diagnosis [16, 18, 19].

To better understand the function of *Lmod2* in vivo, we have identified a *piggyBac* (*PB*) insertional mutant that disrupts *Lmod2* expression and carried out phenotypic characterization of this mutant. We show here that *Lmod2* is crucial for postnatal survival and essential for cardiac function. *Lmod2* deficient mice display DCM with disrupted sarcomeres and intercalated discs (ICDs) including the expression of ICD genes, which offer a new mouse model for this deadly disease.

## Results

### Generation of *Lmod2*-deficient mice

**Fig. 1:**
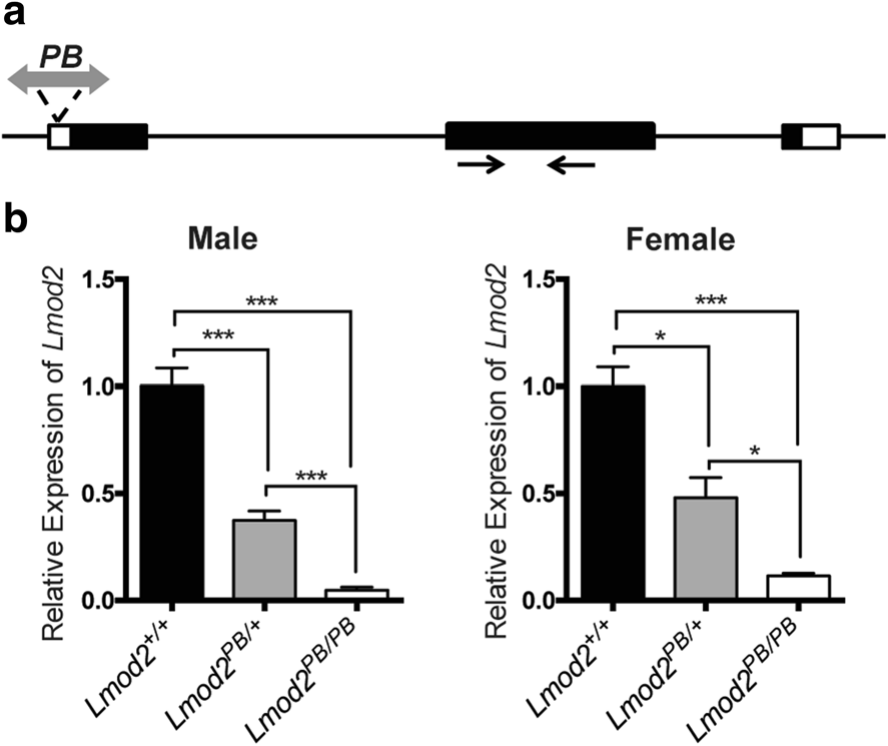
*PB* transposon disrupts *Lmod2* expression. **a** Schematic representation of the *Lmod2* transcription unit and the position of *PB* insertion. Coding and untranslated region are depicted as *black* and *white boxes*, respectively. **b** Quantitative RT-PCR analysis of *Lmod2* mRNA from heart of 3-week-old male and female mice with indicated genotypes, n = 3. *Arrows* PCR primers

Our group has shown that the *PB* transposon is highly active in mice and human cells and could be used to rapidly generate a large collection of insertional mouse mutants in a cost-effective manner [20]. One of the *PB* mutants that we generated has an insertion in the *Lmod2* gene (*Lmod2*^*PB/*+^ in FVB/N background) [21]. This *PB* transposon is inserted in the non-coding region of the first exon of the *Lmod2* transcriptional unit (Fig. 1a) and significantly down regulated the expression of the gene as revealed by quantitative RT-PCR (less than 5 and 10 % in homozygous mutant males and females in comparison to wild-type control Fig. 1b).

**Fig. 2:**
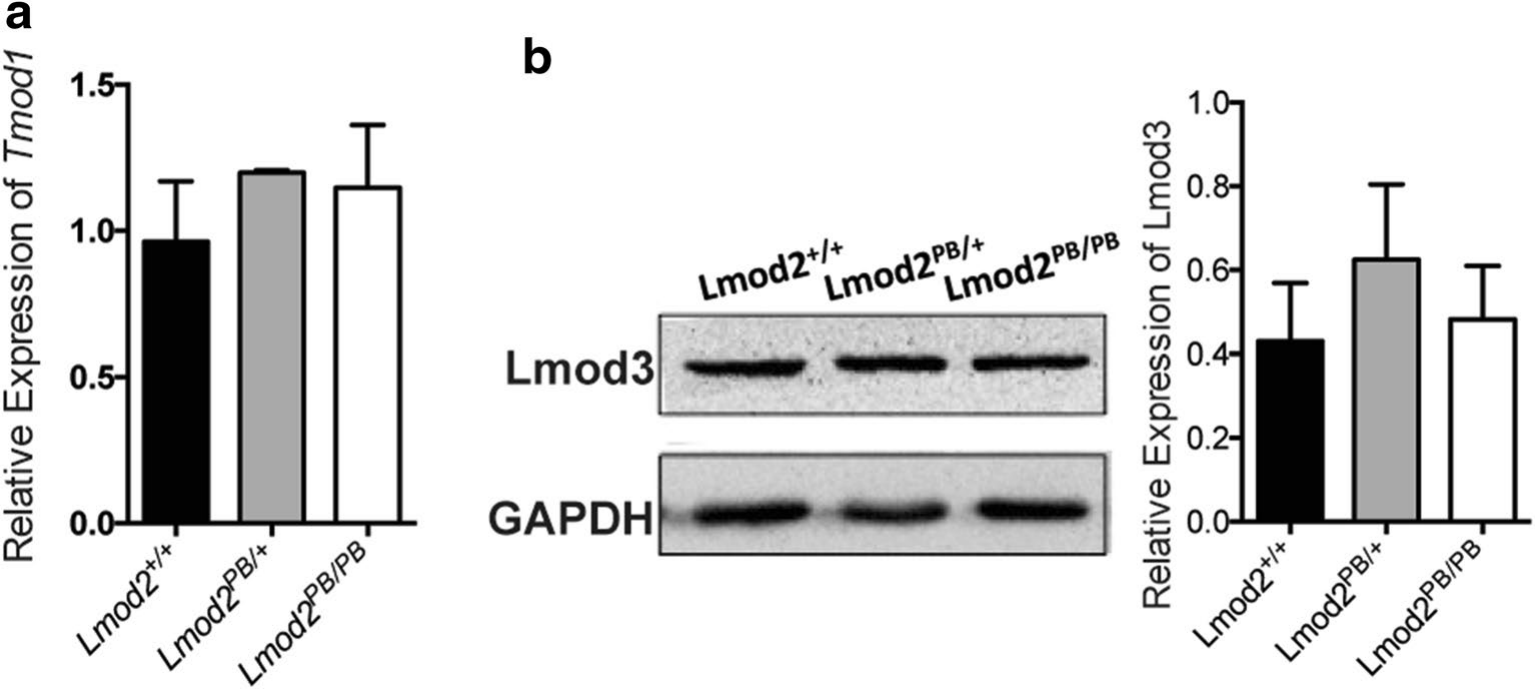
*Tmod1* and *Lmod3* Expression in Lmod2 mutant mice. **a** mRNA level of Tmod1 is not changed in Lmod2^PB/PB^ hearts compared with wild-type control. **b** Lmod3 protein level is also not changed in Lmod2^PB/PB^ mice as shown here in western blotting and statistics

Tmod1 is an antagonist of Lmod2 at the pointed end of the thin filaments in cardiac muscle [5]. It has been suggested that the expression of *Tmod1* could be affected by *Lmod2* [5]. We therefore analyzed the transcriptional level of *Tmod1* by quantitative PCR and found that the mRNA level of *Tmod1* remains the same in 25 days old *Lmod2*^*PB/PB*^ hearts compared to controls (Fig. 2a).

*Lmod3*, another member of the Leiomodin family, also expresses in heart together with *Lmod2*. It might be up regulated to functionally compensate the down-regulation of *Lmod2*. However, Western analysis revealed that the Lmod3 protein level in the hearts of *Lmod2*^*PB/PB*^ is unchanged compared to its controls (Fig. 2b). Together, these data indicate that the phenotypes observed below are likely caused by reduction of *Lmod2* gene activity alone and not a compound effect disrupting other Tmod family members.

### *Lmod2*^*PB/PB*^ mice exhibit postnatal lethality

*Lmod2*^*PB/PB*^ mice are born with the expected ratio as well as their *Lmod2*^*PB/*+^ and wild-type littermates. This indicates that *Lmod2* is not essential for mouse embryonic development.

**Fig. 3:**
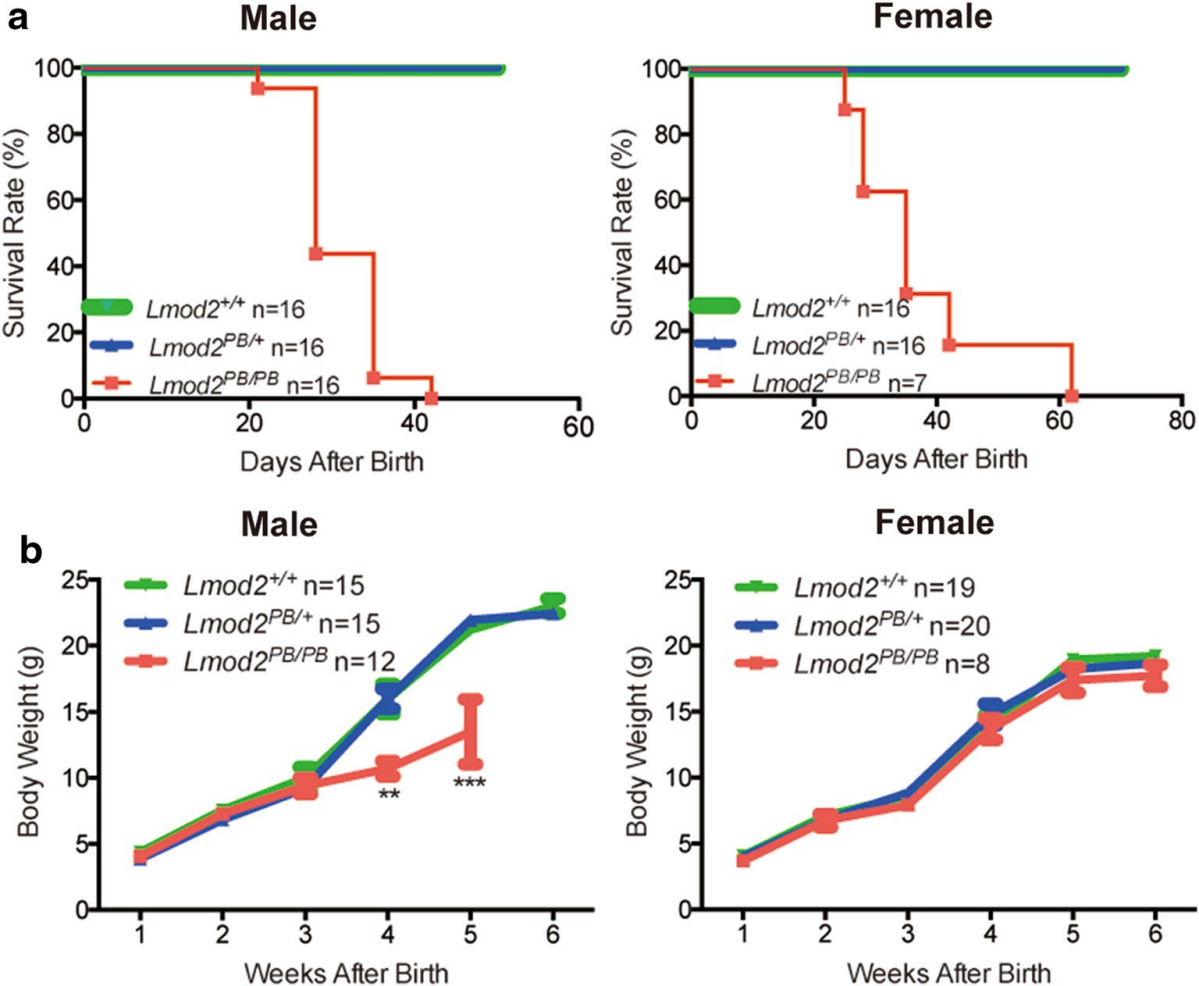
Lmod2^PB/PB^ mice exhibit postnatal lethality and underweight in surviving males. Survival **a** and growth **b** curves of male and female Lmod2^PB/PB^ mice compared with Lmod2^PB/+^ and Lmod2^+/+^ mice

While *Lmod2*^*PB/PB*^ mice are born alive with normal appearance and body weight, the mutant animals exhibit postnatal death around 3rd week of age and are all dead by 9th week (Fig. 3a). Furthermore, male mutant animals are also underweight after three weeks (Fig. 3b). Heterozygous *Lmod2*^*PB/*+^ animals have normal life spans and display no discernable phenotype including fertility. This result indicates that *Lmod2* is crucial for postnatal survival.

### *Lmod2*^*PB/PB*^ hearts display dilated cardiomyopathy defects

The *Lmod2*^*PB/PB*^ mice have no overt signs of distress until 1 to 2 days before death. These signs include curling up, less movement, and dyspnea. Given that Lmod2 is expressed in heart [4], these phenotypes suggest that the mutant animals may suffer from cardiac dysfunction.

**Fig. 4:**
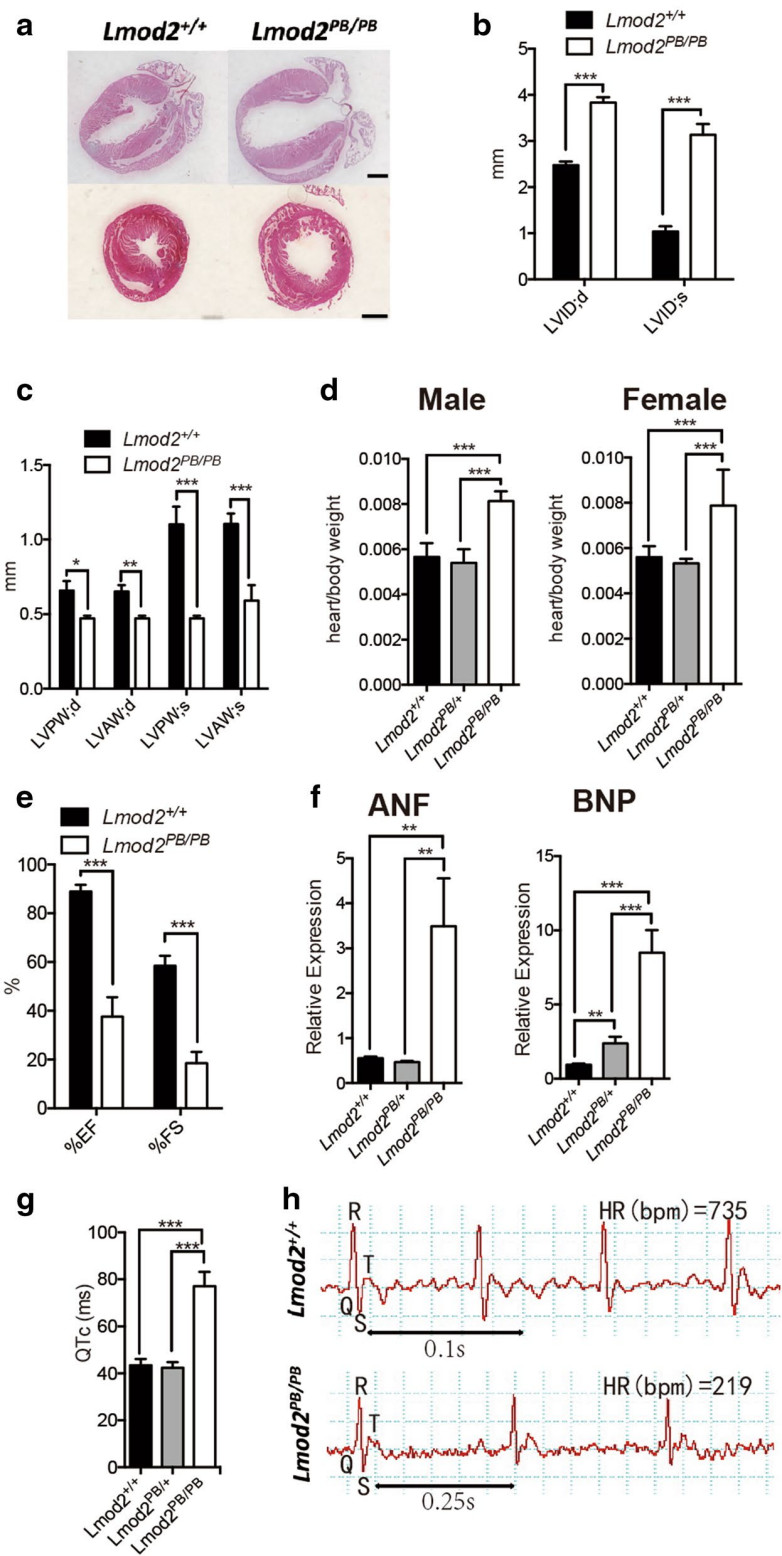
Lmod2^PB/PB^ hearts display dilated cardiomyopathy defects. 25-day old Lmod2^PB/PB^ mice were examined for cardiac morphology and functions. **a** Longitudinal (upper) and transverse (lower) H&E stain sections of paraffin-embedded hearts (*Scale bar* 1 mm). **b**, **c**, **e** Echocardiography analysis of Lmod2^PB/PB^ hearts in comparison to wild-type control n = 5. **b** Higher LV end diastolic and systolic diameter. LVID left ventricular diameter. **c** Thinner LV anterior and posterior wall. LVPW left ventricular posterior wall; LVAW left ventricular anterior wall. **d** Morphometric ratios (heart/body weight) of both male and female Lmod2^PB/PB^ mice are significantly increased. **e** Reduced ejection fraction and fractional shortening values. **f** Quantitative RT-PCR analysis of atrial natriuretic factor (ANF) and brain natriuretic peptide (BNP) revealed increased expression of the heart hypertrophy and heart failure biomarkers n = 3. LV left ventricle; d diastolic; s: systolic. **g**, **h** Electronic Cardiogram revealed lengthening QTc value of Lmod2^PB/PB^ mice in comparison to controls

We therefore conducted histological and physiological analyses of the *Lmod2*^*PB/PB*^ mutant hearts from 25 days old mice before the animals display any signs of distress. Interestingly, all the analyzed *Lmod2*^*PB/PB*^ mutants display enlarged ventricular lumens and thin ventricular walls, phenocopying the symptoms in human DCM patients (n=17, Fig. 4a). The histopathological defects were also confirmed by echocardiography analysis. The mutant hearts had dilated ventricular lumens (Fig. 4b) and thinner heart walls during both systolic and diastolic periods (Fig. 4c). Consistent with these, morphometric ratios (heart weight/body weight) were increased in these animals as well (Fig. 4d).

Echocardiography analysis also revealed that the mutant hearts displayed lower ejection fraction (EF) and fraction shortening (FS) values (Fig. 4e), characteristic DCM deficit. Furthermore, electrocardiography (ECG) analysis revealed lengthened corrected QT interval (QTc) in *Lmod2*^*PB/PB*^ mice in comparison to controls (Fig. 4g, h). The lengthened QTc value means delayed electrical repolarization of the ventricles, indicating ventricular arrhythmia [22], complications that many DCM patients also develop.

The expression of atrial natriuretic factor (ANF) and brain natriuretic peptide (BNP) were examined in mutant animals before signs of heart failure [23]. We discovered that the *Lmod2*^*PB/PB*^ mutant mice already exhibited a significant increase in gene expression for the heart hypertrophy and heart failure biomarkers (Fig. 4f). Finally, Masson’s Trichrome staining revealed that there was no observable increase in fibrosis in these animals (data not shown).

### *Lmod2*-deficiency leads to sarcomere and ICD disorganization

**Fig. 5:**
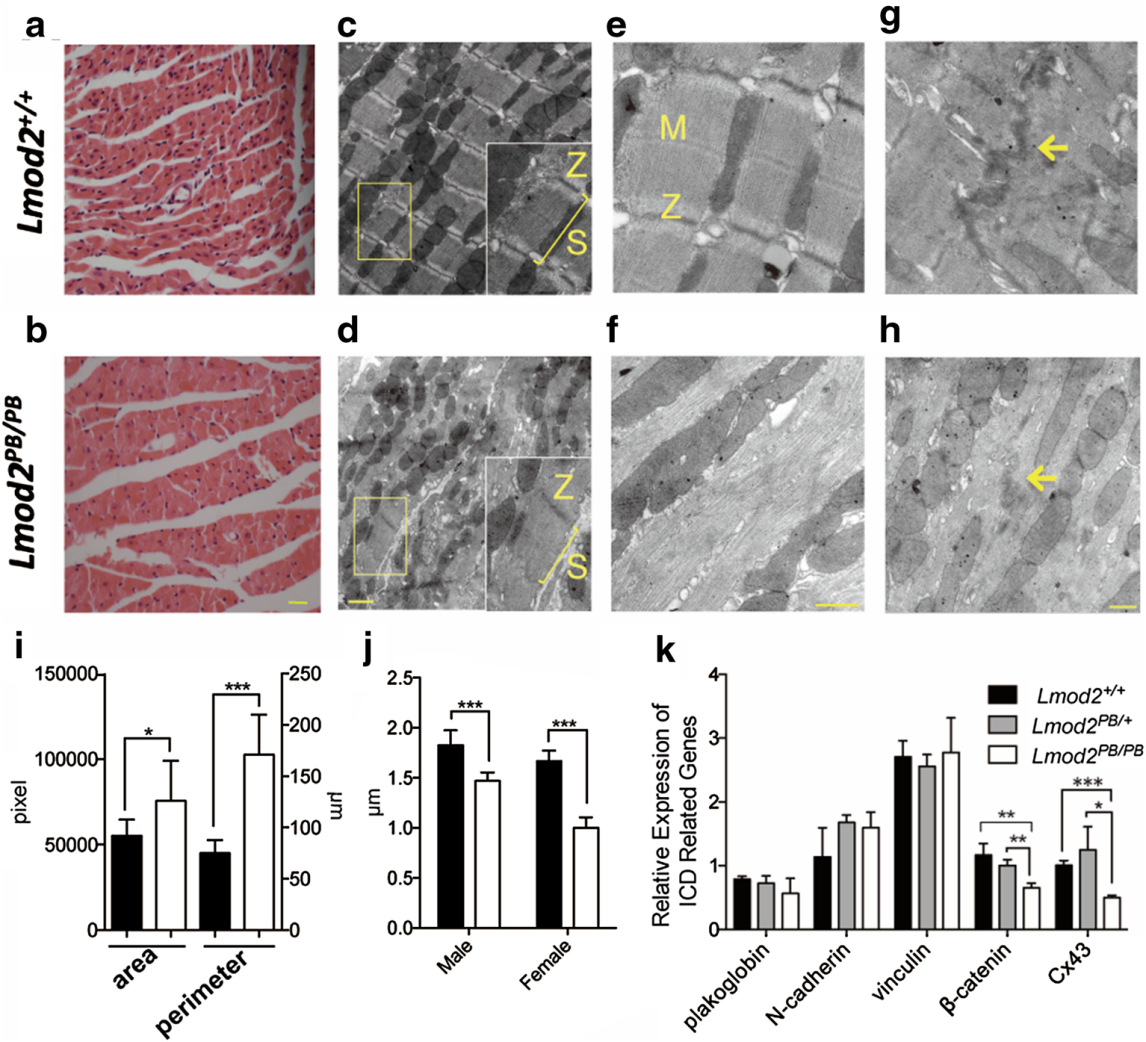
ICD and sarcomere defects of Lmod2^PB/PB^ heart fibers and reduced expression of ICDs proteins. **a**, **b** Longitudinal sections of 25d paraffin-embedded hearts stained with H&E. **c**–**h** Representative electron micrographs of cardiac LV tissue from 25 days old Lmod2^+/+^ (**c**, **e**, **g**) and Lmod2^PB/PB^ (**e**, **f**, **h**) mice. Sarcomeres (S) are shortened and disorganized in Lmod2^PB/PB^ myocardium (**c**, **d**). Z-discs (Z) and M-lines (M) are disorganized and unrecognizable, respectively, in Lmod2^PB/PB^ myocardium (**e**, **f**). Reduced convolution and electron dense of ICD (*arrow*) in Lmod2^PB/PB^ myocardium (**g**, **h**). **i** Area and perimeter of the area between the muscle fibers are increased in Lmod2 ^PB/PB^ hearts compared with wild-type control n = 8. **j** Statistics of sarcomere length in Lmod2^PB/PB^ myocardium n = 20. **k** Quantitative RT-PCR of major intercalated discs (ICD) genes n = 3. *Scale bars* 200 μm in **b**, 1 μm in **d**, 500 μm in **f**, **h**

We further characterized cardiac muscles of *Lmod2*^*PB/PB*^ mice (25 days old) by paraffin section and transmission electron microscope (EM). We noticed that the area between the muscle fibers is larger in *Lmod2*^*PB/PB*^ cardiac muscles than the wild-type control (Fig. 5a, b, i). EM analysis revealed altered sarcomeres with irregular Z-discs and unidentifiable M-lines in *Lmod2*^*PB/PB*^ cardiac fibers (Fig. 5c–f). For those fibers with recognizable Z-discs, measuring the length of the sarcomeres revealed shorter sarcomeres than wild-type control (Fig. 5j). Moreover, the filaments in the *Lmod2*^*PB/PB*^ cardiac fibers are also disarrayed in comparison to wild-type control (Fig. 5e, f). Finally, the ICDs in *Lmod2*^*PB/PB*^ cardiac fibers are less convoluted and have reduced electron dense (Fig. 5g, h). The ICD morphological changes suggest that the mutant fibers might have less ICD proteins. Quantitative RT-PCR (QPCR) indeed identified reduced expression of two of major ICD genes, β-catenin and Connexin43 (Cx43), components of fascia adherens and gap junctions, respectively (Fig. 5k). Together, these data indicate that *Lmod2* deficient cardiac fibers have disrupted sarcomeres and ICDs including the expression of ICD genes.

## Discussion

Using *PB* insertional mutants, we have studied two members of the *Lmod* family, *Lmod2* and *Lmod3*, which express in the cardiac and skeletal muscles. We previously reported that *Lmod3* deficiency lead to severe skeletal muscle weakness with atrophy specific to fast fibers [6]. EM analysis revealed disorganized sarcomeres in skeletal muscles of *Lmod3* mutant mice.

Here we report the characterization of *Lmod2*-deficient mice caused by *PB* insertion in the first exon. Unlike *Lmod3*-deficient mice that are born small with skeletal muscle atrophy [6], *Lmod2*-deficient animals are born with normal appearance and body weight. Only male *Lmod2*-deficient mice exhibit underweight in the 4th week. On the other hand, *Lmod2*-deficient mice exhibit postnatal lethality and all mutant animals die from 3 to 9 weeks after birth. This postnatal lethality together with previous report of *Lmod2* in cultured cardiomyocytes [2, 5] suggests a role of the gene in cardiac function.

We have therefore carried out a pathological analysis of the *Lmod2* mutant heart. Our study shows that the *Lmod2* mutant hearts have enlarged ventricular with systolic dysfunction reflected with EF value less than 50 %, features that are characteristic to DCM patients. Investigation with transmission electron microscopy reveals that the *Lmod2*^*PB/PB*^ cardiac muscles exhibit disordered sarcomeres and ICDs. The morphologies of Z-discs, M-lines, and thin filaments in sarcomeres are all affected.

Intercalated disc is composed of highly organized fascia adherens, gap junctions and desmosomes, and glues cardiomyocytes together. Disruption of ICD would also lead to cardiomyopathy [24]. Recently, it has been shown that mutation in non-ICD component protein could also result in ICD structure abnormality and cardiomyopathy [25]. In the previous reported case, an increase in convolutions of ICD was reported in the cardiomyopathy patient [25]. Here we show that *Lmod2*^*PB/PB*^ cardiomyocytes have a decrease in ICD convolution and cardiomyopathy. Disruption of ICD is also confirmed by reduction in electron density and expression of two of major ICD genes, β-catenin and Connexin43 (Cx43), components of fascia adherens and gap junctions. Therefore, *Lmod2*^*PB/PB*^ mouse model represents a new ICD phenotype related to DCM. During preparation of our manuscript, we noticed that a report of *Lmod2* knockout mouse characterization [26]. While DCM was observed in both models, there were differences in ultrastructure of cardiac muscles. In contrast to *Lmod2*^*PB/PB*^ phenotypes, *Lmod2* knockout model reported widen Z-discs and increased convolutions of ICDs [26]. The difference could be due to the C57BL/6J background for the *Lmod2* knockout and FVB/N for *Lmod2*^*PB/PB*^.

The morphological defects in cardiac muscles proceed the symptom of heart failure in animals. Similarly, the expressions of the heart hypertrophy and heart failure biomarkers, atrial natriuretic factor (ANF) and brain natriuretic peptide (BNP), are elevated before the detection of heart failure. Together, these data show that *Lmod2* deficiency leads to structure abnormality of cardiac muscles, which results in DCM, and that *Lmod2*^*PB/PB*^ mice offer a new model for studying DCM mechanisms and developing therapeutics.

## Conclusion

Taken together, our data reveal that Lmod2 is required in heart thin filaments for integrity of sarcomere and ICD and deficient mice exhibit DCM with ventricular arrhythmias and postnatal lethality. The Lmod2^PB/PB^ mutant offers a valuable resource for interrogation of pathogenesis and development of therapeutics for DCM.

## Methods

### Mouse strains

The founder *Lmod2*^*PB/*+^ mouse was produced by a large-scale genome-wide insertional mutagenesis with the *PB* transposon in the FVB/N mice [20, 27].

### Genotyping

Genomic DNA was isolated by a 56 °C overnight digestion of mouse toes in 200 μL lysis buffer (100 mM NaCl, 10 mM Tris pH 8.0, 25 mM EDTA, and 0.5 % SDS) plus fresh 0.1 mg/ml proteinase K. PCR was performed with primers within exon 1 of *Lmod2* (forward: 5′-AGCTGTCGGCTTTCAATTTTTTTCC-3′; reverse: 5′-TGTCTTCCAGCTCCCTCTCAAG-3′, 247 bp product) and primers within the PB transposon (forward: 5′-CTGAGATGTCCTAAATGCACAGCG-3′ and reverse: 5′-TGTCTTCCAGCTCCCTCTCAAG-3′, 819 bp product). PCR conditions used were as the following: an initial denaturation step at 93 °C for 3 min, then followed by a 40 cycle of 93 °C for 30 s/57 °C for 30 s/65 °C for 2 min, and one more elongation at 65 °C for 10 min as the final step.

### Reverse transcription and quantitative RT-PCR

Total RNA was isolated from 3-week-old mouse heart with Trizol (Invitrogen). The cDNA was synthesized then using the PrimeScript^TM^ RT reagent Kit (Takara). Quantitative RT-PCR was performed with AceQ qPCR SYBR Green Master Mix (Vazyme) according to the manufacturer’s instruction. All quantitative RT-PCR primers are listed in Table 1.

**Table 1:** Quantitative RT-PCR primers

Gene	Forward (5′→3′)	Reverse (5′→3′)
Lmod2	ACCTTATCCCGATTTGCTGAAG	ACCTTGAGCATGTCTGCAATG
GAPDH	TGTTCCTACCCCCAATGTGTCC	GGAGTTGCTGTTGAAGTCGCAG
Tmod1	TGAGCTAGATGAACTAGACCCTG	CGGTCCTTAAATTCCTTCGCTTG
ANF	CATCACCCTGGGCTTCTTCCT	CATCACCCTGGGCTTCTTCCT
BNP	GCGGCATGGATCTCCTGAAGG	GCGGCATGGATCTCCTGAAGG
Plakoglobin	TGGCAACAGACATACACCTACG	GGTGGTAGTCTTCTTGAGTGTG
N-cadherin	AGCGCAGTCTTACCGAAGG	TCGCTGCTTTCATACTGAACTTT
Vinculin	GAGGCTGAACTGCTTCAATCA	CCAGATTTGACGAGGTGCCTA
β-catenin	ATGGAGCCGGACAGAAAAGC	CTTGCCACTCAGGGAAGGA
Cx43	ACAGCGGTTGAGTCAGCTTG	GAGAGATGGGGAAGGACTTGT

### Western blotting

Hearts were dissected and lysed in RIPA buffer with Complete Protease inhibitors (Roche) and PMSF (Phenylmercury acetate) (Sigma) on ice. The BCA Assay (Themo) was applied to quantify total proteins extracted. The western blotting procedure was carried out according to standard protocol with a brief introduction here. Protein bands were separated by SDS/PAGE and transferred to nitrocellulose membranes (Immobilon-P). The transferred membrane was blotted first 5 % skim milk for 1 h. Blots were then incubated with a primary antibody and a secondary antibody consecutively to detect protein of interest. Primary antibodies used are the following: Lmod3 (1:1000; HPA036034, Sigma), GAPDH (1:3000; AC001, ABclonal). Secondary antibody used is the Anti-rabbit IgG antibody conjugated to PerCP-Cy5.5 (1:3000; sc-45101, Santa Cruz). Signal was finally visualized by enhanced chemiluminescence (34,080, Themo).

### Echocardiography and ECG

Mice were anesthetized by inhalation of 1–2 % isoflurane delivered in a gas mixture with oxygen, medical-grade compressed air, and nitrogen. The anesthetized mice went through echocardiography with the Vevo 770 microultrasound system (VisualSonics Inc. Toronto, Canada). Both parasternal long-axis and short-axis views of the left ventricles were analyzed with a 707B transducer according to the manufacturer’s instruction. Data were recorded in M mode.

Conscious and unrestrained mice were used for ECG analysis with the ECGenie electrocardiography system (Mouse Specifics, Inc., Boston, MA) according to the manufacturer’s instruction. Before experimental tests, mice should be trained for 3–5 days to behave normally in this machine. Only data from continuous recordings of 20–30 ECG signals were included in the final analyses. A physiologic waveform analysis platform “eMOUSE” (Mouse Specifics, Inc.) was applied for data analysis.

### Transmission electron microscopy

Samples for transmission electron microscopy were prepared using the standard procedure: hearts were excised, washed in PBS, fixed in 2.5 % glutaraldehyde in 0.1 M cacodylate buffer for more than 2 h, washed in 0.1 M cacodylate buffer for 3 × 15 min, post-fixed with 1 % potassium ferrocyanide reduced OsO_4_ on ice for 2–3 h, dehydrated through graded ethanol and acetone, and finally embedded in EMbed 812 resin. Ultrathin sections (60 nm) were analyzed on a Technai G2 Spirit BioTWIN electron microscope, and images were collected with a 4 × 4 digital camera (Optronics). Length of sarcomere was measured using ImageJ.

### Histology

Hearts were excised, washed in PBS, fixed in 4 % paraformaldehyde solution (Sangon Biotech) overnight, dehydrated, and embedded in paraffin. Longitudinal or transverse sections (10 μm) were stained with a Masson Trichrome Stain Kit (Maixin Biotech) or according to the classical hematoxylin and eosin (H&E) staining protocol (Sigma), and finally mounted with DPX Mountant (Sigma). Light microscopy images were captured using a Nikon Eclipse microscope with a Nikon DS-Ri1 camera and/or a Zeiss Axio Zoom V16 stereomicroscope with an AxioCam MRc camera.

### Statistics

Data were presented as mean ± SED in figures. Two-tailed unpaired Student’s t-test was used unless otherwise stated. The significance is indicated as the label of one or more * with the following categories: (1) * P < 0.05; (2) ** P < 0.01; 3) *** P < 0.005. Prism 6 (GraphPad Software) was used for plotting.

## Abbreviations

ANF: atrial natriuretic factor
BNP: brain natriuretic peptide
CHF: congestive heart failure
DCM: dilated cardiomyopathy
ECG: electrocardiography
EF: ejection fraction
EM: electron microscope
FS: fraction shortening
ICD: intercalated discs
Lmod: leiomodin
Lmod1: leiomodin1
Lmod2: leiomodin2
Lmod3: leiomodin3
LV: left ventricle
LVAW: left ventricular anterior wall
LVID: left ventricular diameter
LVPW: left ventricular posterior wall
PB: piggyBac
QTc: corrected QT interval
Tmod1: tropomodulin1
